# The chemokine network in triple-negative breast cancer: its role in immune microenvironment regulation, and prospects for targeted therapy

**DOI:** 10.3389/fimmu.2026.1883663

**Published:** 2026-08-05

**Authors:** Wanyu Wang, Linhua Chen, Wei Guo, Min Hu, Xuemei Han, Xuan Xia, Wanbin Xu, Wanjun Ma

**Affiliations:** Department of Pharmacy, Union Hospital, Tongji Medical College, Huazhong University of Science and Technology, Wuhan, China

**Keywords:** chemokine, metastasis, targeted therapy, triple-negative breast cancer, tumor immune microenvironment

## Abstract

Triple-negative breast cancer (TNBC) is characterized by high metastatic potential, frequent recurrence and limited targeted regimens. The chemokine network acts as a central orchestrator, reshaping its tumor immune microenvironment (TIME) and determining therapeutic responsiveness. Yet its clinical translation remains hindered by network complexity and insufficient patient stratification. In this review, we systematically synthesize current evidence on how chemokine networks regulate key TIME component (macrophages, T cells, cancer-associated fibroblasts, myeloid-derived suppressor cells, and cancer stem cells) to drive TNBC progression, metastasis, and therapeutic resistance. We also critically evaluate chemokine-targeted interventions, spanning preclinical candidates (small-molecule inhibitors, monoclonal antibodies, miRNA-based therapies, natural products, and nanocarrier systems) to clinical-stage agents. A critical gap emerges from this evaluation: although numerous chemokine axes demonstrate robust preclinical efficacy, most clinical trials have yielded negative or marginal results. These failures are largely attributable to target redundancy, the absence of subtype-specific biomarkers, and suboptimal combination strategies. Based on recent research advances, we propose a three-pillar framework for future therapeutic success: precision subtyping-guided target selection, rational combination regimens (particularly with immune checkpoint inhibitors), and iterative biomarker validation. Overall, this review provides a roadmap for navigating chemokine network complexity, distilling actionable insights for clinical translation, and defining priority research directions to overcome current bottlenecks in TNBC chemokine-targeted therapy.

## 1 Introduction

Breast cancer is subclassified into four molecular subtypes: luminal A, luminal B, human epidermal growth factor receptor 2 (HER-2)-positive, and basal-like (1). The basal-like subtype, more commonly referred to as triple-negative breast cancer (TNBC), is characterized by the absence of estrogen receptor, progesterone receptor, and HER-2 expression. Breast cancer remains a major driver of the global cancer burden. According to GLOBOCAN 2022 estimates, breast cancer accounted for approximately 2.3 million new cases and 670,000 deaths among women worldwide in 2022, with projected figures rising to 3.2 million new cases and 1.1 million deaths by 2050 (2). The National Comprehensive Cancer Network (NCCN) has issued updated clinical practice guidelines for the diagnosis and treatment of breast cancer (3). Per the latest NCCN Breast Cancer Guidelines (Version 5.2025), TNBC treatment now extends beyond conventional chemotherapy or radiotherapy to include programmed cell death ligand 1 (PD-L1) immune checkpoint inhibitors (e.g., pembrolizumab), poly ADP-ribose polymerase (PARP) inhibitors (e.g., olaparib) for targeted therapy. And the antibody-drug conjugate (ADC) sacituzumab govitecan, which has been shown to improve survival for patients with metastatic disease (3, 4). Notably, recent phase III data have suggested that first-line combination therapy with sacituzumab govitecan plus pembrolizumab yields superior outcomes compared with standard chemotherapy plus pembrolizumab in patients with PD-L1-positive advanced or metastatic TNBC (4). Nevertheless, given the highly aggressive nature of TNBC, the development and application of novel targeted drugs remain both a challenge and a central priority in its treatment.

First defined in the 1990s as a distinct class of cytokines, chemokines are chemotactic cytokines that govern the directed migration of immune cells, particularly their recruitment to sites of inflammation or infection (5–9). The first chemokine, chemokine CXCL4—also known as platelet factor-4—was identified by Walz and colleagues in the 1970s. Subsequently studies expanding the chemokine family to include human I-309, interleukin-8 (IL-8), regulated on activation, normal T expressed and secreted (RANTES), macrophage inflammatory protein-2 (MIP-2), among others (10–13). Emerging evidence has further characterized the extensive network of chemokines and their receptors across various cancer types, highlighting their pleiotropic roles in tumor progression, immune regulation, and therapy response (14, 15). Chemokines are now recognized as critical regulators of antitumor immunity, frequently overexpressed within the tumor microenvironment, where they concurrently drive angiogenesis, tumor cell migration, and proliferation (16). Of particular interest in the field is the functional duality of chemokines in oncogenesis: on one hand, they can restrain tumor growth through recruitment of effector T cells; on the other, they may paradoxically promote malignant progression via autocrine or paracrine signaling loops (17).

Following the elucidation of chemokine involvement in cancer and inflammation, the subsequent years witnessed a surge in the development of therapeutics targeting chemokines or their cognate receptors. Maraviroc, a CCR5 antagonist approved by the US Food and Drug Administration (FDA) in 2007 for the treatment of human immunodeficiency virus (HIV) infection, represents the first clinically approved agent targeting a chemokine receptor (18). More recently, a diverse array of novel therapeutic modalities—including antibodies, small molecule inhibitors, microRNAs (miRNAs)—have been actively explored for their potential in TNBC treatment (19–21). Moreover, the development of next generation chemokine inhibitors with improved selectivity and potency holds promise for expanding the therapeutic arsenal against this aggressive malignancy.

This review summarizes the mutual regulatory crosstalk between various chemokines and immune cells in the tumor immune microenvironment (TIME) of TNBC, and elaborates on their pro-tumorigenic or anti-tumorigenic roles throughout TNBC pathological progression. We further highlight their regulatory roles in key stromal components of the tumor microenvironment, including macrophages, T-cells, and cancer-associated fibroblasts. Lastly, we evaluate emerging chemokine-targeting strategies currently under clinical investigation or in preclinical development, and discuss the opportunities and obstacles confronting the translation of these agents into effective TNBC therapies.

## 2 Chemokines

### 2.1 Chemokines and chemokine receptors

Chemokines are a family of small proteins ranging from 8 to 13 kDa in molecular mass, with sequence homology varying widely—from less than 20% to over 90% (22). Interestingly, they share a highly conserved tertiary structure, composed of a disordered N-terminal region of 6–10 amino acids (which contains a critical functional motif common to all chemokines), followed by an N-loop, a triple-stranded β-sheet, and a C-terminal helix. Based on the number and spacing of conserved cysteine residues in the N-terminal region, the chemokine family is classified into four subfamilies: CC, CXC, XC, and CX3C (23), where C denotes cysteine and X represents any amino acid. A unified nomenclature for chemokines and their receptors was formally established in 2000 by the Nomenclature and Standards Committee of the International Union of Basic and Clinical Pharmacology(NC-IUPHAR) subcommittee and has been regularly updated to maintain consistency across the field. Under this system, chemokine ligands are designated with a subfamily prefix, followed by the letter “L” (for ligand) and a sequential number (24). For example, CCL5 formerly known as RANTES, refers to the fifth ligand identified in the CC subfamily (25). Chemokines regulate cell migration, adhesion, and localization by binding to canonical G protein-coupled receptors (GPCRs) or atypical chemokine receptors (ACKRs) (26). To date, the GPCRs family comprises CCR1-CCR10, CXCR1-CXCR6, CXCR8, and CX3CR1, while the ACKR family encompasses ACKR1-ACKR5 (26).

### 2.2 Chemokines and chemokine receptors in tumor

Chemokines exert profound and pleiotropic effects on tumor development, governing key processes including growth, angiogenesis, metastasis, invasion, and immune response. A subset of Chemokines—including CXCL2, CXCL12, CCL20, CCL8, CCL7 and CCL1—has been implicated in promoting tumor malignancy through diverse mechanisms, such as enhancing chemotherapy resistance, proliferation, function of tumor stem cells, immunosuppression, metastasis, and invasion (27–34). Among these, emerging evidence continues to highlight the critical role of the CXCL12/CXCR4 axis in TNBC development, metastasis, angiogenesis, and treatment resistance (35). Moreover, CCL20 has been shown to mediate crosstalk between cancer stem cells (CSCs) and tumor-associated macrophages (TAMs), thereby establishing a pro-tumorigenic microenvironment (36).

Conversely, other chemokines-including CXCL9, CXCL10, CXCL11, CCL2, CCL14, CCL3, CCL4, and CCL7—exert tumor-suppressive effects by enhancing antitumor immune responses, for instance, through promoting CD8^+^ T cells cytotoxicity and facilitating tumor-associated antigen presentation (31, 37–39). Notably, recent studies have developed DPP-4-resistant variants of CXCL9 and CXCL10 that sustain potent antitumor activity, representing a promising avenue for cancer immunotherapy (40).

An additional layer of complexity arises from the observation that the same chemokine can exert opposing effects, depending on the tumor type, disease stage, or secretory site (41–43). For example, in a murine model of breast cancer, CCL2 attenuates lung metastasis by enhancing neutrophil activity. In contrast, in other cancers—including double-negative prostate cancer, esophageal squamous cell carcinoma, and gallbladder carcinoma-CCL2 promotes immunosuppression via recruitment of TAMs, regulatory T cells (Tregs), or myeloid-derived suppressor cells (MDSCs) (44–46). This context-dependent functional duality is also well documented for CX3CL1, which exerts both tumor-promoting and tumor-suppressive effects through its receptor CX3CR1 (47).

## 3 Chemokines and chemokine receptors in the TIME of TNBC

**Figure 1:**
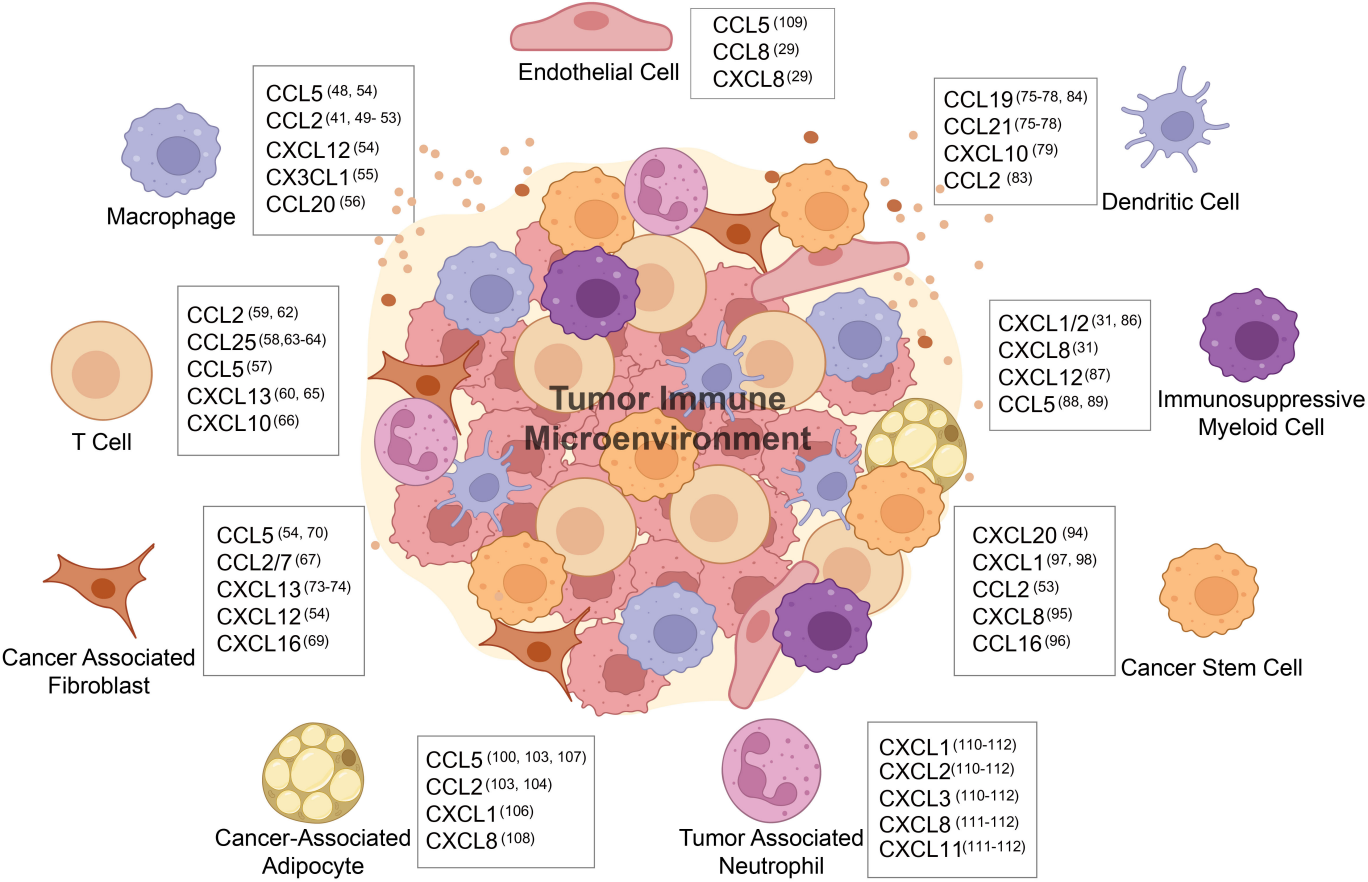
Chemokines associated with multiple cell subsets in the TIME of TNBC. The image depicts the principal cellular populations residing within the TNBC microenvironment, such as TAMs, T cells, CAFs, and CSCs. All chemokines functionally linked to these cell subsets and implicated in TNBC progression are annotated together with their corresponding reference citations. This visually outlines the complex chemokine-governed intercellular crosstalk that modulates TNBC progression. (TIME, Tumor immune microenvironment; TNBC: Triple-negative breast cancer; TAMs, Tumor-associated macrophages; CAFs, Cancer-associated fibroblasts; CSCs, Cancer stem cells).

**Figure 2:**
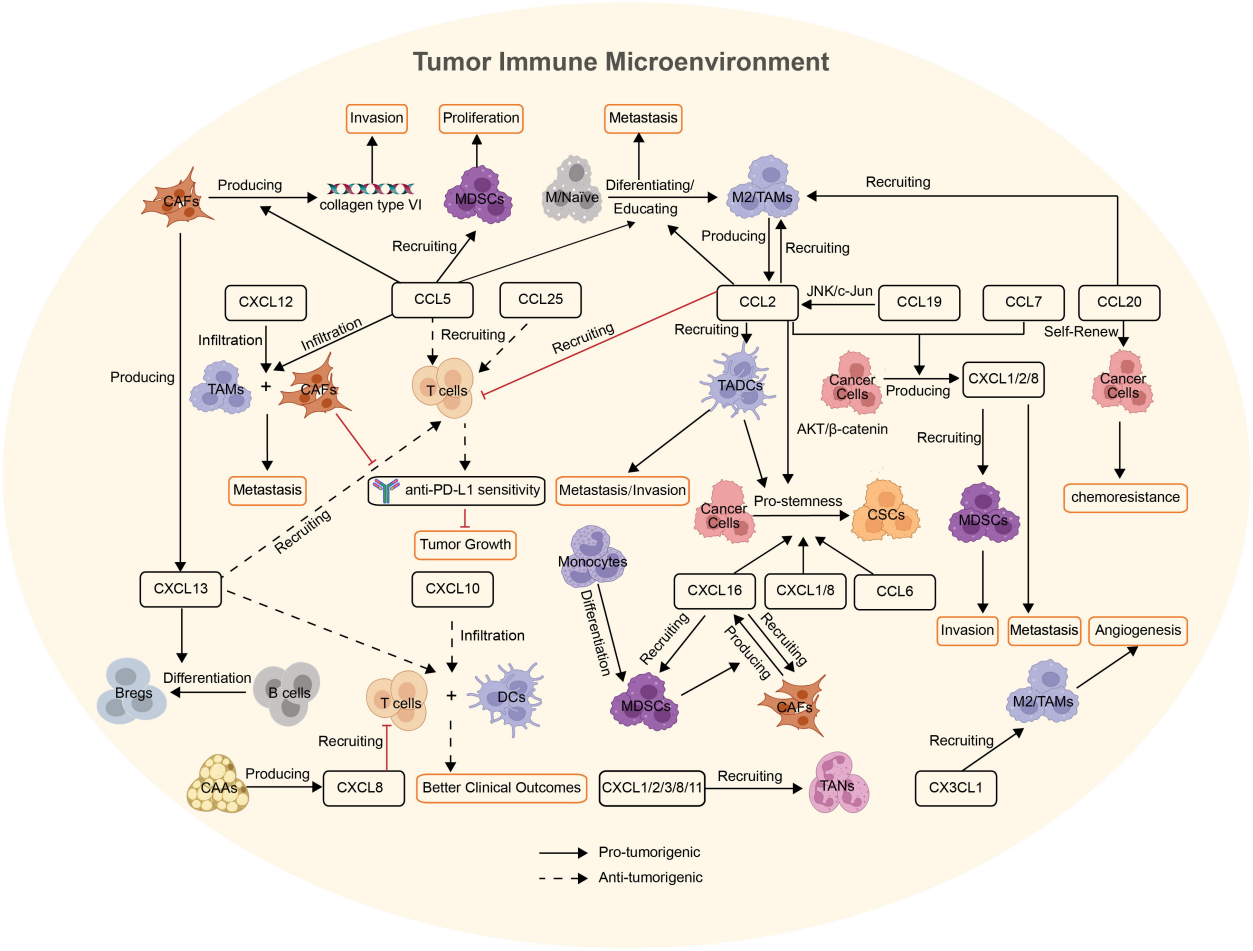
Chemokine-mediated intercellular crosstalk network and its downstream biological effects in the TIME of TNBC. Multiple core chemokines, such as CCL2, CCL5, and CXCL12, serve as signaling mediators to link diverse immune cell populations within the TIME, thereby participating in the progression of TNBC. Solid lines in the image indicate pro-tumorigenic signaling pathways, while dashed lines represent anti-tumorigenic signals. (TIME, Tumor immune microenvironment; TNBC, Triple-negative breast cancer; CAFs, Cancer-associated fibroblasts; TAMs, Tumor-associated macrophages; MDSCs, Myeloid-derived suppressor cells; DCs, Dendritic cells; TADCs, Tumor-associated dendritic cells; CAAs, Cancer-associated adipocytes; Bregs, Regulatory B cells; TANs, Tumor-associated neutrophils; CSCs, Cancer stem cells).

The chemokine–chemokine receptor network within TNBC regulates tumor cells as well as a variety of immune and stromal cells, such as TAMs, MDSCs, T cells, and cancer-associated fibroblasts (CAFs) (**Figure 1**). Relying on diverse molecular mechanisms, this intricate interactive network exerts bidirectional regulatory effects on tumor progression, with both pro-tumorigenic and anti-tumorigenic functions (**Figure 2**). This chapter systematically elaborates the regulatory mechanisms by which various chemokines participate in the initiation and progression of TNBC through immune cell mediation.

### 3.1 Macrophages

Macrophages play a crucial role in TNBC progression and metastasis, largely through their recruitment into the TIME via chemokine signaling. Elevated CCL5 expression promotes the secretion of extracellular vesicles (EVs) that educate macrophages toward a pro-metastatic TAMs phenotype (48). Similarly, upregulated CCL2 correlates with increased lung and bone metastases (49), and has been shown to polarize M0 macrophages toward the M2-TAMs phenotype while recruiting TAMs to enhance the aggressiveness of BRCA1-IRIS Over-expression TNBC cells (41, 50). Recent studies highlight the monocyte CCR2-CCL2 axis as a critical determinant of TAM generation and a potential predictor of response to neoadjuvant therapy in TNBC (51). Moreover, TAMs can also secrete CCL2, thereby accelerating epithelial-mesenchymal transition (EMT) and supporting the tumorigenic capacity of CSCs via the AKT/β-catenin pathway (52, 53). Concurrently, activation of the CCL5/CCR5 and CXCL12/CXCR4 axes enhances the infiltration of TAMs and CAFs (54). Meanwhile, CX3CL1 facilitates angiogenesis by recruiting CX3CR1^+^ macrophages to the tumor microenvironment (TME) (55), while blockade of CCL20 has been shown to significantly reduce macrophage aggregation in MDA-MB-231 cells cultures (56). In brief, CCL2, CCL5, CX3CL1 and CCL20 constitute the core chemokine network governing TAMs in TNBC, which mediate monocyte recruitment, M2 macrophage polarization, angiogenesis, cancer stemness maintenance and tumor metastasis. Accordingly, blocking this chemokine network to suppress the protumor activity of TAMs holds great promise for the clinical treatment of TNBC.

### 3.2 T cells

T cells play a key role in the immune response and can be divided into CD4^+^ T cells and CD8^+^ T cells. Accumulating evidence indicates that chemokines exert profound regulatory control over T-cell function and trafficking within the tumor microenvironment (57–61). Among these, the CCL2-CCR2 axis has been implicated in resistance to PD-L1 blockade, largely through its negative regulatory effects on cytotoxic T lymphocytes; notably, concurrent inhibition of PD-L1 and CCL2 has been shown to overcome such intrinsic resistance in preclinical models (51, 59). Separately, CCL25 has been demonstrated to mediate the chemoattracting CCR9^+^ T cells to the thymus and to potentiate T-cell-mediated immune responses (58). Recent studies have extended these findings: tumor cells engineered to overexpress CCL25 promote intratumoral accumulation of CCR9^+^CD8^+^ T cells, and hydrogel-based intratumoral delivery of CCL25 further augments this infiltration while enhancing the therapeutic efficacy of PD-1 checkpoint inhibitors (62, 63). Moreover, elevated CCL5 levels have been associated with favorable clinical outcomes in TNBC, attributable to enhanced recruitment of activated CD4^+^ T cells, natural killer (NK) cells, M1 macrophages, and CD8^+^ T cells (57). Furthermore, one study found that tumor growth was dramatically suppressed in BALB/c mice injected with 4T1 cells overexpressing CXCL13, an effect mediated by increased recruitment of CD4^+^ and CD8^+^ T cells and dendritic cells (DCs) (CD11b^+^ CD11c^+^) to the tumor nest (60, 64). Additionally, CXCL13^+^ CD8^+^/CD4^+^ T cells have emerged as key antitumor effector subsets in TNBC patients who are sensitive to anti-PD-L1 therapy. Intriguingly, paclitaxel treatment has been reported to deplete these CXCL13^+^ T cells, potentially undermining the antineoplastic function of anti-PD-L1 therapy (61). In parallel, the CXCL10/CXCR3 axis has been identified as a critical regulator of immunological dormancy in TNBC: silencing of CXCL10 reduces CD4^+^ and CD8^+^ T cell infiltration and accelerates tumor onset, whereas a CXCL10-mediated dormancy signature correlates with improved overall survival in TNBC patients (65). Collectively, existing studies demonstrate that chemokines exert differential bidirectional regulation on intratumoral CD4^+^ and CD8^+^ T cells in TNBC, dividing into two distinct signaling branches that mediate immunosuppressive (CCL2/CCR2) and anti-tumor (CCL25, CXCL13, CCL5 and CXCL10/CXCR3) effects respectively. Among them, CXCL13^+^ T cells are critical biomarkers for TNBC patients sensitive to anti-PD-L1 therapy. Notably, paclitaxel impairs CXCL13^+^ T cells and attenuates the anti-tumor efficacy of immunotherapy, indicating that the administration sequence needs to be optimized for combined regimens of taxanes and immune checkpoint inhibitors.

### 3.3 Cancer associated fibroblasts

CAFs play an important role in extracellular matrix remodeling, tumor initiation, metastasis, angiogenesis, and other functions (66). Recent comprehensive reviews have further solidified the recognition of CAFs as crucial mediators in TNBC progression and therapeutic resistance (67). Studies have reported that resident fibroblasts are indispensable participants in TNBC metastasis through chemokines (54, 66, 68–72). For instance, co-culture of adipose-derived stem cells with MDA-MB-231 cells was shown to induce fibroblasts production of pro-oncogenic collagen type VI, while concurrent secretion of CCL5 enhanced cancer cell invasiveness *in vitro (*69). In a related context, Han et al. demonstrated that fibroblasts promote angiogenesis via CCL2/7 secretion, which in turn upregulates tumor cell-derived CXCL1/2/8 and facilitates lung metastasis in TNBC (66). Beyond their direct pro-metastatic effects, CAFs can recruit immunosuppressive cells to shape a pro-tumor TME by producing multiple chemokines, such as CCL2, CCL3, CCL4, CXCL13, and CXCL12 (70). Single-cell transcriptomic analyses have further revealed that antigen-presenting CAFs (apCAFs) as orchestrators of immunosuppressive niches in TNBC via CXCL13-mediated dysregulation of immune cell infiltration, offers both prognostic stratification and novel therapeutic targets (73). Mechanistically, CAFs-derived CXCL13 has also been shown to recruit B cells that differentiate into regulatory B cells (Bregs), leading to an immunosuppressive endpoint in TNBC models (72). Additionally, as described above, stimulation of CCL5/CCR5 and CXCL12/CXCR4 signaling can recruit TAMs and CAFs to TME, promoting lung metastasis in the 4T1 orthotopic mouse model (54). Additionally, CAFs-produced CXCL16 chemoattracts monocytes to the tumor nest, promoting the formation of stroma and inducing more aggressive features of TNBC (68). CAFs-secreted CCL2/5 and CXCL12/13/16 modulate stromal remodeling, angiogenesis and metastasis, and recruit immunosuppressive cells to form a pro-tumor TME. Targeting CAFs-derived chemokines or CAFs subpopulations may overcome TNBC therapeutic resistance and support novel stroma-targeted combination therapies.

### 3.4 Dendritic cells

DCs play a critical role in antigen presentation and initiating immune responses. They can capture antigens and present them to T cells, thereby stimulating anti-tumor responses through chemokines such as CCL19/CCR7 and CCL21/CCR7 (74–77). Elevated CXCL10 levels are associated with the infiltration of DCs and other anti-tumor lymphocytes, and are subsequently related to better clinical outcomes (78). However, dysfunctional or immature DCs are commonly found in cancer patients, which may be associated with tumor suppression (79, 80). Recent studies have uncovered that intratumoral type 1 conventional DCs (cDC1s) in TNBC exhibit defective antigen cross-presentation. Mechanistically, tumor-derived EVs shuttle cytosolic CDC37 into cDC1 endosomes, where it locks antigen-HSP90 binding and prevents antigen translocation to the cytoplasm, thereby impairing cross-presentation and contributing to resistance against immune checkpoint blockade (ICB) (81). Hsu et al. found that MDA-MB-231 cells stimulate tumor-associated DCs (TADCs) to produce CCL2, which in turn recruits TAMs and enhances CSCs function, leading to a pro-tumor condition (82). Besides, CCL19 produced by MDA-MB-231 and MDA-MB-436 cells plays a crucial role in the chemotaxis of DCs through the c-Jun N-terminal Kinase (JNK/c-Jun) pathway (83). DCs recruited via this axis exhibit elevated production of IL-1β, IL-6, and interferon-gamma (IFN-γ), leading to an inflammatory microenvironment that paradoxically supports tumor growth (83). In summary, chemokines mediate bidirectional regulation of tumor immunity by DCs in TNBC. Functional DCs rely on the CCL19/21-CCR7 and CXCL10 signaling axes to accomplish antigen presentation, activate anti-tumor immunity and improve patient prognosis. Nevertheless, the TNBC microenvironment tends to trigger DCs dysfunction: tumor-derived EVs impair the antigen cross-presentation capacity of cDC1s, directly resulting in resistance to immune checkpoint inhibitors. These findings suggest that restoring DCs antigen presentation and blocking DCs-derived pro-tumor chemokines can enhance TNBC responsiveness to immune checkpoint therapy.

### 3.5 Immunosuppressive myeloid cells

Immunosuppressive myeloid cells (IMCs), a heterogeneous population that includes MDSCs, mainly contribute to aggressive tumors by reducing the anti-tumor effects of host immune cells. Li et al. concluded that CX3CR1, CXCR5, CXCR2, CXCR1, CCR7, CCR5, CCR2, and CCR1, which are expressed on MDSCs, are key factors that recruit immune cells to the TME and promote or inhibit intercellular crosstalk (84). At the transcriptional level, activation of the Ras/MAPK pathway has an impact on recruiting MDSCs and further inducing immune evasion via upregulation of CXCL1, CXCL2, and CXCL8 in TNBC cells (31, 85). Given the role of MDSCs, combined targeting of CXCR2 (the CXCL8 receptor) or the Ras/MAPK pathway alongside T-cell–reinvigorating immunotherapies may represent a rationally justified therapeutic strategy for TNBC patients (31). The CXCR4/CXCL12 axis drives MDSCs-mediated immunosuppression. In TNBC mouse models, treatment with balixafortide, a CXCR4 antagonist, in combination with anti-PD-1, significantly reduced tumor growth and extended survival, decreasing T-cells exhaustion and MDSCs signatures while increasing tertiary lymphoid structures (86). Ban et al. said CCL5-deficient myeloid precursors lack membrane CCR5 and are unresponsive to tumor-derived CCL5. This myeloid alteration increases CD8^+^ T cells and decreases Tregs in tumor-draining lymph nodes (87). miRNAs are non-coding RNAs that bind to the 3′UTRs of target genes to modulate tumor progression. Gu found miR-9 facilitates the chemotactic accumulation of G-MDSCs via the SOCS3/CCL5/CCR5 axis, thereby accelerating breast cancer proliferation (88). Hence, disrupting the coordinated chemokine axes that drive MDSCs recruitment represents a promising strategy to reverse immunosuppression and enhance the antitumor efficacy of immunotherapy in TNBC.

### 3.6 Cancer stem cells

CSCs are increasingly recognized as critical drivers of tumor progression, owing to their self-renewal capacity, which underpins tumor initiation, drug resistance, cancer recurrence, and metastasis (89, 90). In TNBC, CSCs pose particular clinical challenges, and chemokines signaling has been implicated in their pro-tumorigenic functions (91, 92). For instance, Chen et al. demonstrated that CCL20 is markedly upregulated in response to taxane-based chemotherapy, promoting breast cancer stem cells (BCSCs) renewal and enhancing chemoresistance in TNBC (93). Similarly, downregulation of CCL2 has been associated with reduced CSCs frequency, correlating with diminished tumor growth and metastasis burden (53). In parallel, overexpression of IL-8 in tumor cells expanded the CXCR1^+^ CSCs population and augmented their self-renewal ability, thereby accelerating tumor growth and metastasis in TNBC xenograft models (94). The chemokine CCL16 has also been implicated in sustaining CSCs properties via the CCR2/GSK3β/β-catenin/OCT4 signaling axis, a mechanism that may account for the positive correlation between elevated CCL16 expression and accelerated tumor proliferation observed in TNBC (95). Studies also found that CXCR2 expression was detected in 4T1 cells exhibiting prominent CSC characteristics, and BCSCs could self-renew and proliferate through autocrine CXCL1-CXCR2 signaling (96, 97). Collectively, multiple chemokine axes cooperatively regulate CSCs stemness, chemoresistance, and tumor progression, positioning these axes as attractive therapeutic targets for eliminating BCSCs in TNBC.

### 3.7 Other cells: cancer-associated adipocytes, endothelial cells, tumor associated neutrophils

#### 3.7.1 Cancer-associated adipocytes

Mammary adipose tissue constitutes the primary component of the breast cancer TME. Normal adipocytes undergo reprogramming to transform into cancer-associated adipocytes (CAAs) (98). CAAs secrete bioactive molecules including CCL2, CCL5 and CXCLs, which act as intercellular messengers to remodel tumor cell phenotypes and modulate TNBC invasion and metastasis (98–104). Ruan et al. demonstrated that co-culturing mature adipose tissue with TNBC cells activates STAT3/NF-κB p65 signaling, which upregulates CXCL1 release in TNBC cells and IL-6 secretion in adipocytes. This reciprocal crosstalk drives TNBC progression (105). Another study based on three-dimensional co-spheroid models demonstrated that direct co-culture of TNBC cells with adipose-derived stromal cells or adipocytes upregulates CCL5/CCR1 expression and enhances the migratory capacity of MDA-MB-231 cells (106). Huang et al. demonstrated that CXCL8 secreted by CAAs remodels the TME by inhibiting CD4^+^ and CD8^+^ T cell infiltration, thereby driving TNBC cell proliferation, EMT and metastasis (107).

#### 3.7.2 Endothelial cells

In the TME, endothelial cells (ECs) are also enriched and act as neoplastic factors. Zhang et al. demonstrated that tumor-derived PAI-1 stimulates ECs to release CCL5. And CCL5 promotes TNBC cell migration and invasion while increasing PAI-1 secretion from tumor cells, creating a positive feedback loop to accelerate TNBC metastasis (108). Besides, studies have demonstrated that co-culture of TNBC MDA-MB-231 cells with endothelial colony-forming cells (ECFCs) promotes CCL8 secretion from ECFCs. The elevated CCL8 further induces IL-8 production in MDA-MB-231 cells to augment tumor invasive aggressiveness (29). Clinically, high CXCL8 expression is closely correlated with poor recurrence-free survival in TNBC patients (29).

#### 3.7.3 Tumor associated neutrophils

Accumulating evidence reveals that tumor associated neutrophils (TANs) exert pro-tumorigenic functions to facilitate malignant progression. One study verified that TNBC-secreted CXCR2-binding GRO chemokines (CXCL1/2/3) potentiate TAN motility and recruit these neutrophils into the TME (109). Tumor necrosis factor-related apoptosis-inducing ligand (TRAIL) activates the NFKB2 pathway in tumor cells, promotes the secretion of CXCL1/2/3/8/11 and IL-6, recruit TANs and induces tumor immune suppression. Such TANs can inhibit T cell proliferation and upregulate the immunosuppressive phenotype of Tregs (110). Nevertheless, Li et al. found anti-tumor phenotype of TANs: CXCR2-dependent neutrophil infiltration strengthens CD8^+^ T cell activity and suppresses pulmonary metastasis, which correlates with favorable patient prognosis (111).

Collectively, CAAs, endothelial cells and TANs jointly shape an immunosuppressive TME via distinct chemokine axes to govern TNBC invasion and distant metastasis; context-dependent dual functions of TANs also provide novel cell-targeted therapeutic insights for TNBC intervention.

Overall, various cell subsets in the TIME of TNBC do not regulate tumor progression independently. Instead, they form a highly interconnected cell communication network with chemokines as signaling mediators, jointly mediating dual pro-tumorigenic and anti-tumor effects. As shown in **Figure 2**, CAFs secrete the chemokine CXCL13 to activate systemic anti-tumor immune responses via recruiting T cells. Macrophage-secreted CCL2 activates the AKT/β-catenin signaling pathway and endows tumor cells with stem cell properties. Meanwhile, CCL2 further recruits TADCs and macrophages, continuously amplifying cancer stemness and markedly boosting tumor invasion and metastasis. In addition, monocytes within the microenvironment can differentiate into MDSCs with potent immunosuppressive activity. MDSCs induce CAFs to produce CXCL16, while CXCL6 reciprocally recruits CAFs and MDSCs to continuously expand immunosuppressive cell populations and consolidate the immunosuppressive TME.

This intricate chemokine crosstalk network also illustrates the bottlenecks encountered in current clinical treatment of TNBC, which accounts for the unsatisfactory efficacy of single-agent immunotherapy in most patients. Accordingly, when investigating the tumor-regulating functions of chemokines, co-culture systems containing multiple immune cell types should be established to recapitulate the authentic *in vivo* immune microenvironment to the greatest extent. Meanwhile, experiments with combined intervention of multiple chemokines need to be designed to systematically dissect the overall regulatory patterns of chemokine networks, thereby providing more credible and sufficient experimental evidence for the development of chemokine-targeted therapies against TNBC.

## 4 Chemokines and chemokine receptors in TNBC treatment

As detailed above, chemokines govern multiple facets of TNBC progression through diverse and interconnected mechanisms. In response, substantial efforts from both academic institutions and the pharmaceutical industry have been directed toward the development of chemokine-targeted therapeutics and the design of novel treatment strategies for TNBC patients. Herein, we summarize the latest advances in chemokine-targeting drug candidates and emerging therapeutic approaches, with the aim of providing a comprehensive overview of current progress in TNBC treatment and offering practical guidance for clinical translation. **Tables 1** and **2** summarize representative agents currently under preclinical evaluation and those in active clinical trials, respectively.

**Table 1:** Preclinical studies of chemokine candidates in TNBC.

Category	Target chemokines/chemokine receptors	Drugs/promising agents	Tumor model/cell lines	Effects	Combination treatment	PMID
Inhibitor/Antagonist	CXCR4	IT1t	MDA-MB-231 zebrafish model	Inhibit early metastasis		26744352
		4F-benzoyl-TN14003	Tail vein injection of MDA-MB-231 in SCID mice	Decrease lung metastasis		12935890
		CTCE-9908	Intravenous (tail vein) or intracardiac injection of MDA-MB-231 in athymic mice	Inhibit tumor growth and metastasis to lung and bone		19212637; 19482312
		AMD3100	Dorsal flank injection of MDA-MB-231 in nude BALB/c mice	Enhance sensitivity to carboplatin	Carboplatin	22016821
		AMD3100	Human xenograft athymic nude mice with MDA-MB-231	Enhance sensitivity to radiation/tumor growth delay	Radiation	29317253
		AMD3100	NOD/SCID mouse model (MDA-MB-231)	Inhibit tumor growth and metastasis	Olaparib (PARP1 inhibitor)	36209974
		liposomal-ADM3100/Plerixafor	Murine TNBC model (4T1)	Prolong survival time	anti-PD-L1	33131745
		polypeptide TAT-DV1-BH3	Nude mice model (MDA-MB-231)	Inhibit tumor growth and metastasis		26527862
		CXCR4-scFv-RBM/miRNA nano-complexes	BALB/c mouse model (4T1)	Inhibit tumor growth	miR-127-5p	31719921
		anti-CXCR4-DPC/miR-34a	Orthotopic MDA-MB-231 tumor models	Anti-metastasis	miR-34a	32735880
		POL5551	NOD-scid-IL2R gamma^null^ mouse model (MDA-MB-231)	Anti-metastasis	Eribulin	26269605
		silibinin	MDA-MB-231 cells	Inhibit migration and invasion		25172795
	CCL5	CCL5-Ab/CCL5-pep	MDA-MB-231 cells	Inhibit invasion		27027351
		leronlimab	Athymic nude model (MB-MDA-231-Luc2-eGFP)	Inhibit lung metastasis	Doxorubicin	33485378
		maraviroc	Nude rat model (MDA-MB-231)	Inhibit bone metastasis		30456574
		maraviroc	Athymic nude mouse model (MDA-MB-231-luc-D3H2LN)	Inhibit tumor growth and metastasis	cMR16-1 (an anti-IL-6 receptor antibody)	29898755
		N-[4-(Benzyloxy)-3-methoxybenzyl)]adamantane-1-amine (DZH2)	MDA-MB-231 cells	inhibit tumor migration		39468982
		peptide (no name)	MDA-MB-231 cells/ZNF451 KnockOut Mouse	inhibit TAMs recruitment and activation		37342906
	CXCR1	Reparixin	Nude BALB/c mouse model (MDA-MB-231)	Inhibit brain metastasis	Paclitaxel	26517518
	CXCR2	CXCR2 inhibitor	Nude BALB/c mouse model (MDA-MB-231)	Inhibit tumor growth	Bevacizumb (an antibody of anti-VEGF)	33520697
		SB225002	Female BALB/c nude mice model (4T1)	Inhibit metastasis		40188812
		AZD5069	MDA-MB-231 cells	Enhance anti-tumor effects of doxorubicin/atezolizumab	Doxorubicin/Atezolizumab (an inhibitor of PD-L1)	35517812
	CXCL8	HuMax-IL8	SCID mouse model	Inhibit MDSCs recruitment and EMT		29093275
	CCL25/CCR9	NP-siCD47/CCL25	BALB/c mouse model (4T1)	Inhibit tumor growth and metastasis	Anti-PD-1/PD-L1	32064335
	CCL20	CCL20 antibody	Nude BALB/c mouse model (MDA-MB-231)	Inhibit bone metastasis		28851919
	CCR7	CCR7 trap	Murine 4T1 Breast Cancer model	Inhibit lymphatic metastasis		30690891
miRNA	CCR2	CCL16 shRNA	NOD/SCID mouse model (MDA-MB-231)	Inhibit the initiation of tumor		33500726
	CCL18	microRNA-181b	MDA-MB-231 cells	Inhibit proliferation, migration and invasion		28105154
	CCL8	miRNA let-7a	MDA-MB-231 cells	Inhibit proliferation and migration		26898455
	CCL21-CCR7	microRNA let-7a	Zebrafish embryo model (MDA-MB-231)	Inhibit invasiveness		22251626
	CXCR4	miR-381	MDA-MB-231 cells	Inhibit proliferation, EMT, and metastasis		28012397
	CCL2	CCL2-Ca-TAT/siRNA	Nude BALB/c mouse model (MDA-MB-231)	Inhibit tumor growth and metastasis		27283985
Natural compunds and other agents	CCL5	Emodin	Nude BALB/c mouse model (MDA-MB-231)	Inhibit tumor growth, and lung or liver metastasis		29693154
	CXCR4	Thymoquinone	NCr-Foxn1nu mouse model (MDA-MB-231)	Inhibit multiple lung, brain, and bone metastases		30564115
		Prostratin	MDA-MB-231 cells	Exhibit cytotoxicity to cancer cells		29435066
		Ginsenoside Rg3	MDA-MB-231 cells	Inhibit migration		21455623
		Norepinephrine	MDA-MB-231 cells	Inhibit invasion		25912576
		Saikosaponin A	athymic BALB/c nude mouse model (MDA-MB-231)	Inhibit metastasis		32047724
		Recombination protein P21	MDA-MB-231 cells	Inhibit proliferation and invasion		33195200
		si-CrkL	MDA-MB-231 cells	Inhibit invasion		25476480
		Z-guggulsterone	MDA-MB-231 cells/BALB/c nude mouse model (MDA-MB-231 cells)	inhibit migration and invasion		37290260
		Triterpenes of Prunella vulgaris	MDA-MB-231 cells/nude mouse model (MDA-MB-231 cells)	inhibit proliferation, migration and invasion; promote apoptosis		40076586
	CXCL1	Pentagalloyl glucose	MDA-MB-231/MDA-MB-468 cells	Inhibit proliferation		33707603
		XIAOPI formula	Mice and zebrafish xenotransplantation models (MDA-MB-231)	Enhance taxol chemosensitivity	Taxol	31629951

Tumor necrosis factor-related apoptosis-inducing ligand (TRAIL); Mesenchymal stem cells (MSCs); Myeloid-derived suppressor cells (MDSCs); Severe combined immunodeficiency mouse model (SCID mouse model); Cancer stem cells (CSCs); Epithelial-mesenchymal transition (EMT); Vascular endothelial growth factor (VEGF).

**Table 2:** Clinical trials involving chemokine candidates for TNBC treatment.

Target	Molecule	Phase	Study design	Cancer type	Status	Country	Identifier
CCR8	S-531011/S-531011+ Pembrolizumab	Phase Ib/II	Non-Randomized; Open Label; Sequential Assignment	Advanced or Metastatic Solid Tumors	Recruiting	United States; Japan	NCT05101070
	BAY3375968/BAY3375968+Pembrolizumab	Phase I	Non-Randomized; Open Label; Sequential Assignment	NSCLC/TNBC/HNSCC/Gastric cancer/Melanoma	Active, Not Recruiting	United States; Belgium; Canada; China; France; Singapore; Spain; United Kingdom	NCT05537740
	TAK-188	Phase I/II	Non-Randomized; Open Label; Sequential Assignment	Advanced or Metastatic Solid Tumors	recruiting	United States	NCT07205718
	LM-108+Toripalimab+Eribulin/LM-108+Toripalimab+Nabpaclitaxel	Phase II	Non-Randomized; Open Label; Parallel Assignment	Recurrent or Metastatic TNBC	Recruiting	China	NCT06387628
	Denikitug(GS-1811)/Denikitug+Zimberelimab	Phase I	Non-Randomized; Open Label; Sequential Assignment	Advanced Solid Tumors	Recruiting	United States; Australia; Canada; Spain; Taiwan	NCT05007782
	CHS-114/CHS-114+Toripalimab	Phase I	Randomized; Open Label; Parallel Assignment	Advanced Solid Tumors; HNSCC	Recruiting	United States	NCT05635643
	BGB-A3055/BGB-A3055+Tislelizumab	Phase I	Non-Randomized; Open Label; Single Group Assignment	Advanced or Metastatic Solid Tumors	Active, not recruiting	United States; Australia; China; France; South Korea	NCT05935098
	CHS-114+Toripalimab	Phase I	Randomized; Open Label; Parallel Assignment	Advanced or Metastatic Solid Tumors	Recruiting	United States; Taiwan	NCT06657144
	BMS-986340/BMS-986340+Nivolumab(BMS-936558-01)	Phase I/II	Non-Randomized; Open Label; Parallel Assignment	CCA/GC/MS-CRC/NSCLC/HNSCC/RCC/UC/PAAD/Melanoma/Ovarian Neoplasms/TNBC	Recruiting	United States; Australia; Canada; China; Germany; Israel; Italy; Japan; Spain;	NCT04895709
	CM369	Phase I	Non-Randomized; Open Label; Sequential Assignment	Advanced Solid Tumors and Hematologic Malignancies	Recruiting	China	NCT05690581
	Denikitug (GS-1811)/Denikitug (GS-1811)+Zimberelimab	Phase I	Non-Randomized; Open Label; Sequential Assignment	Advanced Solid Tumors	Recruiting	United States; Australia; Canada; Spain; Taiwan;	NCT05007782
	LM-108/LM-108+Anti-PD-1 Antibody	Phase I/II	Non-Randomized; Open Label; Sequential Assignment	Advanced Solid Tumor	Terminated	United States	NCT05255484
CCL2	CNTO888+Docetaxel/CNTO888+Gemcitabine/CNTO888+Paclitaxel+Carboplatin//CNTO888+pegylated liposomal doxorubicin	Phase I	Non-Randomized; Open Label; Single Group Assignment	Solid Tumors	Completed	United States; Spain	NCT01204996
	CNTO888	Phase I	Non-Randomized; Open Label; Single Group Assignment	Cancer	Completed	No location data	NCT00537368
CXCL8 (IL-8)	HuMax-IL8	Phase I	Open Label; Single Group Assignment	Advanced Malignant Solid Tumors	Completed	United States	NCT02536469
	Nivolumab (Anti-PD-1) + BMS-986253 (Anti-IL-8) + SBRT	Phase I	Open Label; Single Group Assignment	Multiple Metastases in Advanced Solid Tumors and Melanoma	Recruiting	United States	NCT04572451
CXCL12/CXCR4	MB1707	Early Phase I	Open Label; Single Group Assignment	Advanced Cancer (Advanced Solid Tumor/Breast Cancer/NSCLC/OC/Pancreatic Neoplasms)	Withdrawn	not provided	NCT05465590
CCR5	leronlimab (PRO 140)	Phase II	Open Label; Single Group Assignment	CCR5+ Locally Advanced or Metastatic Solid Tumors	Completed	United States	NCT04504942
	Leronlimab+Carboplatin	Phase I/II	Non-Randomized; Open Label; Parallel Assignment	TNBC	Terminated	United States	NCT03838367
	Leronlimab (PRO 140)	not provided	not provided	CCR5+ Metastatic TNBC	No longer available	not provided	NCT04313075
	leronlimab (PRO 140)	not provided	Non-Randomized; Open-Label	Metastatic TNBC	Available	United States	NCT07536815
	OB-002	Phase I	Non-Randomized; Open Label; Sequential Assignment	Metastatic Cancer (Colorectal Cancer/Pancreatic Cancer/Gastric Cancer/Breast Cancer/Urothelial Carcinoma	Withdrawn	not provided	NCT05940844
CXCR1/2	Reparixin	Phase II	Open Label; Single Group Assignment	Breast Cancer	Terminated	United States;	NCT01861054
	Reparixin+Paclitaxel	Phase Ib	Non-Randomized; Open Label; Single Group Assignment	Metastatic Breast Cancer	Completed	United States	NCT02001974
	Reparixin+Paclitaxel/Paclitaxel+Placebo	Phase II	Randomized; Triple Blind (Participant Investigator Outcomes Assessor); Parallel Assignment	Metastatic Breast Cancer	Completed	United States; Belgium; Czechia; France; Italy; Poland; Spain	NCT02370238
	SX-682+M7824+MVA-BN-CV301	Phase I/II	Non-Randomized; Open Label; Sequential Assignment	Advanced Solid Tumors	Completed	United States	NCT04574583
CXCR4	MB1707	Early Phase I	Open Label; Single Group Assignment	Advanced Solid Tumor (Breast Cance/NSCLC/OC/Pancreatic Neoplasms)	Withdrawn	not provided	NCT05465590
	X4P-001	Phase I/II	Open Label; Single Group Assignment	TNBC	Unknown status	China	NCT05103917
	LY2510924+Durvalumab	Phase I	Non-Randomized; Open Label; Parallel Assignment	Solid Tumor	Terminated	not provided	NCT02737072
	Balixafortide(POL-6326) +Eribulin/Balixafortide+Nabpaclitaxel	Phase Ib/II	Non-Randomized; Open Label; Sequential Assignment	HER2-negative breast cancer in the advanced setting, with any ER and PgR status	Withdrawn	not provided	NCT04826016
	POL-6326	Phase I	Open Label; Single Group Assignment	HER2 negative and Metastatic Breast Cancer	Completed	United States; Spain	NCT01837095
	MB1707	Phase I	Open Label; Single Group Assignment	Advanced Cancer	Withdrawn	No location data	NCT05465590
	X4P-001+Toripalimab	Phase Ib/II	Open Label; Single Group Assignment	TNBC	Unknown status	China	NCT05103917
	LY2510924+Durvalumab	Phase Ia/Ib	Non-Randomized; Open Label; Parallel Assignment	Solid Tumors	Terminated	No location data	NCT02737072
	Balixafortide+Eribulin/Eribulin	Phase III	Randomized; Open Label; Parallel Assignment	Metastatic Breast Cancer, Locally Recurrent Breast Cancer	Terminated	United States; Argentina; Belgium; Brazil; Czechia; France; Italy; Russia; South Korea; Spain; Taiwan; Ukraine; United Kingdom	NCT03786094

Cervical cancer (CCA); Gastric/Gastroesophageal junction adenocarcinoma (GC); Microsatellite stable (MS)-colorectal cancer (CRC); Non-small-cell lung cancer (NSCLC); Head and neck squamous cell carcinoma (HNSCC); Renal cell carcinoma (RCC); Urothelial carcinoma (UC); Pancreatic adenocarcinoma (PAAD); Triple-negative breast cancer (TNBC); Ovary cancer (OC).

### 4.1 Preclinical studies involving chemokine targets for TNBC treatment

In recent years, a substantial number of chemokine-related targets have been investigated in the context of TNBC. Herein, we summarize the key preclinical findings and discuss their therapeutic implications.

#### 4.1.1 Candidates for directly targeting chemokines or chemokine receptors

##### 4.1.1.1 CXCR4/CXCL12

Targeting the CXCR4/CXCL12 axis has demonstrated substantial anti-tumor efficacy in multiple hematological and solid malignancies. Numerous inhibitors have been developed to disrupt this signaling pathway, including IT1t, TIQ-15, AMD3100 (plerixafor), T140) and monoclonal antibodies including MSX-122, BMS-936564. For instance, both IT1t and 4-F-benzoyl-TN14003 (a T140 analog) have been shown to effectively suppress metastasis in TNBC (112, 113). Similarly, the CXCR4 antagonist CTCE-9908 has been found to reduce metastasis burden and restrain tumor growth; however, it does not significantly affect the reduction of metastasis events (114, 115). Beyond its direct blocking effects, AMD3100 enhances chemosensitivity to Carboplatin by regulating IL-1 expression from MSCs and increases radiosensitivity by promoting G2-M cell-cycle arrest and apoptosis (116, 117). Additionally, Lu et al. found that the liposomal delivery of AMD3100 could enhance its efficacy by targeting its ligand and locking CXCR4 expressed on the cell membrane (118). In a complementary finding, Misra et al. reported that targeted nanocarriers are selectively internalized by CXCR4-positive cells and effectively disrupt CXCR4-mediated signaling, corroborating CXCR4 as a viable target for nanocarrier-based therapies (119). TAT-DV1-BH3, a fused polypeptide that functions as another CXCR4 antagonist, significantly inhibits tumor growth through down-regulation of matrix metallopeptidase 9 (MMP9) expression and inhibition of phosphoinositide 3 kinase (120). Furthermore, Wang et al. showed that Silibinin (a CXCR4 antagonist) can compete with CXCL12 for receptor binding, thereby blocking the activation of downstream Akt and Erk pathways and inhibiting the migration and invasion of cancer cells (121). A dual-targeting peptide-drug conjugate based on CXCR4 and folate receptor alpha (FOLR1) has been developed to deliver monomethyl auristatin E (MMAE) into tumor cells. Notably, this conjugate also remodels the immunosuppressive TME by enhancing intratumoral CD8^+^ T cells infiltration and reducing MDSCs (122).

Studies have shown that combinatorial strategies targeting the CXCL12/CXCR4 axis yield significant therapeutic benefits in TNBC. For instance, the combination of AMD3100 and PARP1 inhibitor has shown superior antitumor efficacy effects in BRCA-proficient TNBC relative to CXCR4 inhibitors alone (116, 117, 123, 124). And the combination of liposomal-AMD3100 and anti-PD-L1 antibodies conferred marked advantages in the treatment of TNBC (118). In another preclinical study, POL5551, a CXCR4 antagonist, when combined with eribulin, significantly prolonged survival and reduced metastatic dissemination in TNBC mouse models compared with eribulin monotherapy (125). Interestingly, the combination of CXCR4 inhibitors and miRNA has also shown promising outcomes. CXCR4-scFv-RBM/miRNA nano-complexes, a good unit of CXCR4 targeting single-chain variable fragment (scFv) and miR-127-5p (an important miRNA for polarizing M1 macrophages), exerted a tumor inhibition effect accompanied by favorable biosafety and enhanced drug-loading capacity in TNBC mouse model (19, 126). Yang et al. discovered that the Dextrin-PEI-CM nano-complex (DPC) could carry both miR-34 and the anti-CXCR4 cyclam monomer (CM). The resulting DPC/miR-34a complex not only abrogates CXCR4 but also counteracts metastasis driven by upregulated CD44 expression, further potentiating apoptotic cell death in cancer cells (127). Overall, the CXCR4/CXCL12 axis is a key therapeutic target in TNBC. Multiple inhibitors have been developed, including AMD3100, CTCE-9908, POL5551, and silibinin. Single-agent CXCR4 blockade shows limited efficacy. Combination with PARP inhibitors, anti-PD-L1, or eribulin enhances therapeutic outcomes. Nano-formulations and miRNA co-delivery further improve targeting and efficacy. These strategies highlight the potential of CXCR4/CXCL12 as a combinatorial target.

##### 4.1.1.2 CCL5/CCR5

CCL5 up-regulation promotes lymphatic angiogenesis, as well as tumor metastasis and proliferation. CCL5-Ab (a CCL5 mono-antibody) or CCL5-pep (a CCL5 receptor peptide) can strongly inhibit the pro-tumorigenic function of CCL5 (99, 128). Leronlimab, a CCR5-targeting antibody has been shown to prevent lung metastasis and enhance the DNA damage effect of Doxorubicin in TNBC (129). Zhang et al. found a peptide that disrupts the ZNF451-SLUG interaction, this intervention downregulated CCL5 expression, which in turn impairs the migration and activation of TAMs, ultimately suppressing TNBC progression (130). Maraviroc, an additional CCR5 antagonist, can inhibit the colony formation and proliferation of cancer cells, as well as reduce bone metastasis in a TNBC mouse model (131). Verteporfin, a selective CCR5 antagonist identified by high-throughput screening, suppresses TNBC growth and metastasis via the CCR5-YAP1 axis independent of its photodynamic effect. Mechanistically, this axis critically regulates chemokine expression and promotes EMT (132). Furthermore, N-[4-(Benzyloxy)-3-methoxybenzyl] adamantane-1-amine (DZH2), a dual CCR5/CXCR4 inhibitor, reduced the migration of MDA-MB-231 cells to only 4%, demonstrating an inhibitory potency that surpassed that of maraviroc by over 100-fold (133). In addition, the combination of maraviroc and cMR16-1 (an anti-IL-6 receptor antibody) significantly attenuated both tumor growth and TNBC metastasis (134, 135). At the mechanistic level, the CCL5/CCR5 axis has also been shown to orchestrate the chemotactic recruitment of G-MDSCs via miR-9-mediated targeting of SOCS3 (88). Together, these findings show that the CCL5/CCR5 axis promotes angiogenesis, metastasis, and proliferation in TNBC. Multiple inhibitors have been developed, including CCL5-Ab, leronlimab, maraviroc, verteporfin, and DZH2. These strategies underscore CCL5/CCR5 as a promising therapeutic target.

##### 4.1.1.3 CXCR1/2/3

Recent studies have reinforced the therapeutic value of CXCR1/2 inhibition in TNBC. Han et al. reported that a novel CXCR2 inhibitor, when combined with bevacizumab (an anti-VEGF antibody), suppressed tumor growth in MDA-MB-231 xenograft model via modulation of the HEYL-CXCL1/2/3 axis (136). Similarly, Reparixin, a CXCR1 inhibitor, when used in combination with paclitaxel, can significantly inhibit brain metastasis by preventing the activation of CSCs and enhancing apoptosis (94). Besides, another study revealed that AZD5069, a CXCR2 antagonist, not only counteracted doxorubicin-induced transforming growth factor-β (TGF-β) upregulation but also enhanced the cytotoxicity of the PD-L1 inhibitor atezolizumab (137). Moreover, pharmacological blockade with SB225002, an inhibitor of CXCR2, was shown to elicit apoptosis in TNBC cells and reduce metastasis burden in xenograft models, confirming CXCR2 as a viable target for TNBC therapy (138). Nevertheless, intratumoral plasmid IL-2 can induce the chemokine CXCR3 gene signature in tumor cells, which is advantageous for antigen delivery, CD8^+^ T cell expansion, and anti-PD1 therapeutic immunosensitivity (139). Collectively, CXCR1/2 inhibition shows promising therapeutic potential in TNBC by targeting CSCs, enhancing chemosensitivity, and modulating the TIME.

##### 4.1.1.4 other chemokines or chemokine receptors

A monoclonal antibody, CCR7 mAb, has the potential to reduce cancer metastasis; however, studies indicate that inhibiting CCR7 on DCs or lymphocytes could lead to immunosuppression (140, 141). To circumvent this limitation, An’s team designed a nanoparticle delivery system capable of carrying a CCR7 trap (a plasmid encoding a CCR7 antagonist) to the tumor site. Their results indicated that the CCR7 trap could access the tumor nest and significantly suppress lymphatic metastasis without eliciting detectable immunosuppression in TNBC model (142). In a parallel approach, Shen et al. designed a trap targeting CXCL13, which similarly yielded favorable outcomes in TNBC patients (72). Nanoparticle delivery system has also been successfully applied to target chemokines for the treatment of TNBC. For example, the NP-siCD47/CCL25 complex, which carries small interfering RNA (siRNA) for CD47 and the CCL25 chemokine, can effectively increase the infiltration of CCR9^+^ T cells and augment the antitumor activity of anti-CD47 therapy in a TNBC mouse model (58). Intriguingly, this NP-siCD47/CCL25 platform also synergizes with PD-1/PD-L1 checkpoint blockade, highlighting its potential as a component of novel combinatorial regimens for TNBC (58). In a complementary strategy, an injectable, thermo-responsive CCL25-loaded hydrogel facilitates sustained intratumoral delivery, promoting dose- and time-dependent infiltration of CCR9^+^CD8^+^ T cells into the TME and markedly augmenting the efficacy of PD-1 inhibitors (63).

HuMax-IL8, an antibody of CXCL8, prevented the recruitment of MDSCs and inhibited the EMT transformation of cancer cells (143, 144). Moreover, HuMax-IL8 has been shown to reverse the mesenchymal phenotype in claudin-low TNBC models and, when combined with docetaxel, significantly decreases PMN-MDSCs recruitment at the tumor site (145). Concurrent inhibition of IL-6 and IL-8 has been demonstrated to significantly suppress tumor growth in a TNBC mouse model (146). Furthermore, inhibition or neutralization of IL-8 can significantly enhance the chemosensitivity to paclitaxel and potentiate the antitumor efficacy of bortezomib in TNBC (147, 148). Additionally, the human antigen R-regulated chemokine CCL20 also exhibits an anti-neoplastic effect in the MDA-MB-231 mouse model (149). More interestingly, a study reported in 2020 revealed that peptides functionally mimicking CXCL12-CXCL4 heterodimers inhibit TNBC cell migration (150). Overall, CCR7, CXCL13, CCL25, and CXCL8 represent emerging targets, with nanocarriers, traps, and hydrogels enabling precise delivery. CCR7 inhibition remains challenging due to immunosuppression risks. CCL25/CCR9 and CXCL8 blockade, particularly in combination with ICIs, show translational promise.

#### 4.1.2 Candidates for targeting chemokines or chemokine receptors via miRNA

It is well established that CCL18 released by TAMs plays a crucial role in breast cancer metastasis (151). MicroRNA-181b can suppress the proliferation, migration, and invasion of MDA-MB-231 cells by downregulating CCL18 expression (20). And short hairpin RNA (shRNA)-mediated silencing of CCL16 significantly decreased CSCs characteristics and suppressed tumor initiation through regulation of CCR2/GSK3β/β-catenin/OCT4 axis in TNBC (95). Another miRNA, let-7a, can reverse the pro-tumor effect exerted by CCL8 by downregulating Lin28 levels and inducing G2/M arrest in the TNBC cell line (152). In a complementary study, Kim et al. demonstrated that let-7a binds to the 3’UTR of CCR7, thereby blocking the CCL21-CCR7 pathway and inhibiting invasiveness in a zebrafish embryo model (153). In a distinct approach, the incorporation of RNA triple-helix structures (including the tumor suppressor miRNA-205 and the oncomiR inhibitor miRNA-221) and CXCR4-targeting siRNA duplexes, was shown to confer enhanced specificity and selectivity for TNBC treatment (154, 155). Similarly, miR-381 can target CXCR4 and inhibit its effects on proliferation and metastasis (156). Fang et al. developed a novel strategy to target CCL2 utilizing calcium-mediated cross-linking between siRNAs and the trans-activating transcriptor (TAT) peptide. The complex of Ca-TAT/siRNA can inhibit the renewal of CSCs and recruitment of M2 macrophages *in vivo*, which demonstrated a favorable approach for clinical treatment of TNBC for the first time (53). Collectively, miRNAs target chemokines or chemokine receptors to suppress metastasis, CSCs properties, and immune recruitment, highlighting miRNA-based therapeutics as a promising strategy for TNBC.

#### 4.1.3 Candidates for targeting chemokines or chemokine receptors through natural compounds

Song et al. reported that Emodin can inhibit the EMT process and metastasis by repressing the production of CCL5 by adipocytes in TNBC (157). Thymoquinone, on the other hand, was found to downregulate CXCR4 by inhibiting the interaction between NF-κB and the CXCR4 promoter, thereby blocking the effects of CXCL12 on migration and invasion (21). Similarly, Z-guggulsterone also exerts antitumor activity by targeting CXCR4 through the same mechanism (158). Triterpenes of Prunella vulgaris significantly suppressed the proliferation, migration, and invasion of MDA-MB-231 cells, and induced dose-dependent apoptosis by targeting the PTP1B/PI3K/AKT/mTOR and IL-24/CXCL12/CXCR4 signaling pathways (159). The natural polyphenol Gossypol also exhibited anti-tumor effects by downregulating CCL2 and IL-8 (160). Honokiol decreases CCL2 expression and suppress M2 macrophage polarization via the STAT6/STAT3 pathways, while also reducing macrophage recruitment through the CCL2/CCR2 axis to prevent lung metastasis in TNBC (161). Prostratin demonstrated cytotoxic effects on MDA-MB-231 cells via the SIK3/CXCR4 pathway (162). Ginsenoside Rg3 was found to exert anti-migration effects on cancer cells by inhibiting CXCR4 (163). Saikosaponin A, a compound derived from Radix bupleuri, also exhibited anti-TNBC effects by modulating CXCR4 expression (164). A recent comprehensive review summarized the potential of polyphenolic natural compounds, such as Curcumin, Resveratrol, Gossypol, Cardamonin, Butein, Quercetin, and EGCG-in modulating the TIME through inhibition of NF-κB pathway and CCL2 chemokine signaling in TNBC (165). Finally, Pentagalloyl glucose (PGG), a polyphenolic natural compound, has been associated with the prevention of numerous cancers. PGG can decrease the expression of GRO-α/CXCL1, which is negatively correlated with the proliferation rate of MDA-MB-231 and MDA-MB-468 cells (166). Natural compounds broadly target chemokine axes including CCL5, CXCR4, CCL2/CCR2, and CXCL1 to suppress metastasis and modulate the immune microenvironment, underscoring their potential as chemokine-directed therapeutics.

#### 4.1.4 Candidates for targeting chemokines/chemokine receptors through other factors

The XIAOPI formula is an anticancer drug commonly used in the management of patients at elevated risk of breast cancer. Wang et al. discovered that this formula could enhance the chemosensitivity of taxol by modulating the CXCL1/HMGB1-induced autophagic pathway, while demonstrating a favorable safety profile in TNBC animal models (167). A subsequent preclinical study further showed that XIAOPI suppresses chemoresistance and metastasis by inhibiting EV/CXCL1-induced TAM/PD-L1 signaling (168). Beyond the context of this formulation, CXCL12 has been implicated as a malignant regulator for tumor progression by activating the CrkL adapter protein (169). Borges et al. discovered that the recombination protein P21 could substitute for CXCL12 and interact with CXCR4, resulting in reduced proliferation and lower invasive activity in MDA-MB-231 cells (170). Another study also found that norepinephrine could decrease the CXCR4 level and inhibit MDA-MB-231 invasion through engagement of the β2-adrenergic receptor (171). Collectively, beyond direct targeting of chemokines and their receptors, upstream regulatory molecules, pharmacological interventions and signaling pathway modulation can indirectly silence chemokine signaling axes, offering diverse therapeutic strategies for TNBC. These findings overcome the limitations of single-target inhibitors and provide critical experimental evidence for developing low-toxicity, broad-spectrum combination regimens and novel adjuvant agents targeting chemokines against TNBC.

### 4.2 Clinical trials involving chemokine targets for TNBC treatment

Several chemokine targets are currently under active clinical trials. As summarized in **Table 2**, several candidates are being investigated for TNBC treatment, including those targeting CCR5, CCR8, CCL2, CXCR1, CXCR4, and CXCL8 (172). Leronlimab (PRO 140), a CCR5 antagonist, is being evaluated in phase I/II trials for TNBC. The results of three clinical trials (NCT04504942, NCT03838367, NCT04313075) suggest that leronlimab is well-tolerated, with no serious drug-related adverse events reported. Additionally, monotherapy has demonstrated evidence of antitumor activity, associated with a significant improvement in 1-year overall survival (OS) (173). Recently, the expanded access program (NCT07536815) has also been opened to provide leronlimab to patients with advanced mTNBC who lack alternative satisfactory treatment options. BAY3375968, an anti-CCR8 antibody, has been posted for a clinical trial (phase I) which is recruiting TNBC patients for treatment with BAY3375968 alone or in combination with pembrolizumab. Preclinical data indicate that BAY3375968 mediates potent depletion of CCR8^+^ human Tregs *in vitro*, driven by antibody dependent cell-mediated cytotoxicity (ADCC) and antibody dependent cellular phagocytosis (ADCP) mechanisms. Consequently, this effect inhibits the suppressive function of Tregs on CD8^+^ T cells, thereby unleashing their tumor-killing immune activity and mediating an antitumor response (174). In addition to BAY3375968, eight anti-CCR8 antibodies are either in active clinical recruitment or have not yet entered the stage, including S-531011, TAK-188, LM-108, GS-1811, CHS-114, BGB-A3055, BMS-986340, and CM369 (172). However, a Phase I/II clinical trial of LM-108, an anti-CCR8 antibody, has been terminated for undisclosed reasons.

Besides, clinical development of Reparixin, a CXCR1/2 inhibitor has failed to demonstrate clear clinical value across three breast cancer trials. An early window-of-opportunity study (NCT01861054) was terminated prematurely due to slow patient accrual, but available data indicated that Reparixin monotherapy was well-tolerated and associated with reduced expression of CSCs markers (175). A phase Ib trial (NCT02001974) showed that Reparixin combined with weekly paclitaxel achieved an objective response rate of 29.6%, providing preliminary support for further evaluation (176). However, the pivotal phase II trial (NCT02370238) failed to meet its primary endpoint. Adding reparixin to paclitaxel did not significantly improve progression-free survival or overall survival compared with paclitaxel alone in patients with metastatic TNBC (177). Although the next-generation oral CXCR1/2 inhibitor SX-682 demonstrated acceptable safety profile in Phase II trials, it failed to demonstrate meaningful clinical activity in advanced solid tumors, resulting in the termination of further development. These results led to the termination of CXCR1/2 inhibitor clinical development in breast cancer, suggesting that single-agent blockade of the CXCR1/2 pathway may not yield meaningful therapeutic benefits in this disease setting.

Furthermore, clinical exploration of CXCR4 pathway antagonists in TNBC and solid tumors remains in its early stages, with limited overall efficacy signals, and most pivotal trials have concluded with negative results or early termination. Studies on the oral CXCR4 inhibitor X4P-001 are still in the early stage; however, its clinical status is currently undisclosed, and no data have been publicly released yet. Although the peptide antagonist Balixafortide (POL6326) demonstrated acceptable safety and preliminary survival signals in a phase I trial in combination with eribulin (178), the subsequent phase III FORTRESS trial (NCT03786094) failed to validate its clinical benefit, with neither objective response rate nor progression-free survival (PFS) superior to single-agent chemotherapy, leading to the termination of its development for breast cancer indications. The phase I combination trial of LY2510924 with durvalumab (NCT02737072) was terminated early due to strategic adjustments, providing only safety data without evidence of meaningful anti-tumor activity (179). The phase I trial of the novel conjugate drug MB1707 was withdrawn prior to initiation, with no supporting clinical data available. Overall, strategies involving single-agent CXCR4 blockade or combination with existing therapies have not translated into significant clinical benefits in breast cancer and solid tumors, suggesting that the therapeutic value of this pathway remains to be further validated. Future efforts may necessitate more refined patient stratification or novel combination regimens to circumvent current efficacy limitations.

Finally, CXCL8 and CCL2 represent additional clinically explored targets. For example, HuMax-IL8 (BMS-986253), an IL-8 antibody, has been evaluated in phase I/II clinical to confirm its safety and determine the maximum tolerated dose (180). The results indicated that HuMax-IL8 is a well-tolerated monoclonal antibody with acceptable safety, but it has limited anti-tumor activity, and subsequent development may shift to combination therapy with PD-1 inhibitors (180). With respect to CCL2 blockade, Brana et al. evaluated Carlumab, a CCL2 monoclonal antibody. Although the study identified a safe dose, Carlumab proved ineffective at sustaining the suppression of serum CCL2 levels, leading the investigators to recommend against its clinical use (181). Another clinical trial reached the same conclusion (182). These findings indicate limited clinical value for anti-CCL2 antibody-based therapies. Notably, no recent updates regarding Carlumab in TNBC have emerged, and its development for oncological indications was discontinued owing to insufficient efficacy.

In summary, clinical trials targeting chemokine axes in TNBC have yielded largely disappointing results. CCR5 blockade with leronlimab showed acceptable safety and modest survival signals but failed to demonstrate robust efficacy. CXCR1/2 inhibitors (reparixin, SX-682) and CXCR4 antagonists (balixafortide, LY2510924) failed to meet primary endpoints across multiple trials. Similarly, IL-8 and CCL2 antibodies also showed limited antitumor activity. These failures are largely attributable to target redundancy, suboptimal patient selection, and insufficient combination strategies. Future success will require refined biomarker-driven trial designs and rational combination regimens.

## 5 Discussion

### 5.1 The complexity of chemokine networks and the immune microenvironment

Firstly, the chemokine system is complex, comprising over 50 different chemokines and approximately 20 different chemokine receptors. This complexity is further compounded by extensive ligand–receptor promiscuity: a single chemokine can bind to more than one receptor, and multiple chemokines might share the same receptors. For example, CCL2 can not only bind to CCR2 but also interact with CCR4. Moreover, the CCR2 receptor can recognize more than one ligand, including CCL2, CCL6, CCL7, CCL8, CCL12 and CCL13 (183–190). The sheer complexity and overlapping interaction networks within this system have impeded a comprehensive understanding of its functional roles in tumor biology, while the overlapping interactions between chemokines and their receptors are often described as promiscuous (191). Beyond chemokine signaling axes, Duan and colleagues identified a core copper-driven oncogenic cascade, demonstrating that TNBC progression is orchestrated by multilayered regulatory factors (192). Therefore, targeting a single specific chemokine or receptor to inhibit or enhance its effects presents a substantial therapeutic challenge (193). Secondly, the functional landscape of chemokines or their receptors within the TME is characterized by cooperative and often synergistic interactions. For example, CXCL1/2/8 and CCL2/7 are involved in a positive feedback loop between fibroblasts and tumor cells, playing crucial roles in promoting tumor progression (66). Therefore, this cooperative behavior renders the identification of broadly effective, universal inhibitors particularly challenging. The intricate crosstalk between CSCs and TAMs, which is critically mediated by chemokines signaling has been extensively documented, further highlighting the complexity of these interaction networks (194, 195).

Lastly, chemokines exert dichotomous functions within TME: they can orchestrate anti-tumor immune response by recruiting effector immune cells, yet simultaneously promote the recruitment of some tumorigenic immune cells and facilitating the establishment of a pro-tumorigenic milieu (196). This context-dependent duality has been systematically integrated for several chemokines. For instance, CCL7 both facilitates progression by shaping the TME and enhances anti-tumor immune responses by recruiting T cells and NK cells; similarly, CCL2 has been shown to promote tumor progression and exerts antitumor activities by activating immune surveillance (14, 197). Furthermore, numerous chemokine receptors are expressed on both tumor cells and normal cells, rendering selective targeting of tumor cells challenging. As an illustrative example, CCL3 or CCL4 secreted by B cells can recruit anti-tumor TILs to the TME by binding to CCR1 and CCR5 (198–202), but they can also paradoxically accelerate tumor growth when produced by TAMs, MDSCs, or MSCs (203–209). Thus, chemokine-targeting drugs with high cell-type selectivity are critically needed to abrogate the pro-tumorigenic functions of chemokines or their receptors while preserving their anti-tumor functions.

Emerging intervention strategies include: 1) Blockade of immunosuppressive chemokine signaling using small-molecule inhibitors or neutralizing antibodies; 2) Delivery of immunostimulatory chemokines via advanced platforms such as hydrogels and oncolytic viruses; 3) genetic engineering CAR-T cells to overexpress specific chemokine receptors for improved tumor homing (210). Consequently, a deeper understanding of the complex chemokines in the TME, along with the development of more selective and targeted drugs, will be essential to fully realize the potential of chemokine-targeted therapies.

### 5.2 Heterogeneity of TNBC

TNBC is a heterogeneous disease comprising several subtypes that differ in their molecular and clinical characteristics, and chemokines critically modulate this heterogeneity, driving tumor progression, metastasis, and therapy resistance (211). Burstein et al. analyzed RNA and DNA profiles from 198 TNBC tumor samples and identified four subtypes of TNBC: luminal-AR, mesenchymal, basal-like immune-suppressed, and basal-like immune-activated (212). This foundational framework was subsequently extended by Lehmann et al., who established an early subtyping system for TNBC, while more recent advances in multi-omics and single-cell technologies have substantially refined our understanding of tumor heterogeneity (213–215). In parallel, a molecular subtyping system developed by the Fudan University Shanghai Cancer Center has been pivotal in translating transcriptomic subtyping into clinical practice. Through integrative multi-omics analyses encompassing single-cell RNA-seq, bulk transcriptomics, epigenomics, and mutational profiling the research team delineated three molecular subtypes (CS1, CS2, and CS3), of which CS3 exhibits prominent chromatin remodeling and high proliferative activity, suggesting specific therapeutic vulnerabilities (216).

Recent single-cell and spatial transcriptomic analyses have provided unprecedented resolution of TNBC heterogeneity. In a landmark study analyzing pre-treatment tissue samples from 101 treatment-naïve TNBC patients, researchers revealed four tumor archetypes and 13 metaprograms, and further mapped 49 immune/stromal states. Notably, macrophage subtypes and interferon-, HLA-, and cell-cycle-related metaprograms (not T cells alone) were found to be the primary determinants of chemotherapy response (217). Deep learning further classified TNBC into three subtypes and identified CXCL9 as a biomarker for ICB response, which is regulated by indoleamine 2,3-dioxygenase 1 (IDO1) (218). In addition, non-coding RNA-mediated m6A reprogramming also modulates organ-specific metastatic tropism across distinct TNBC subtypes. Integrated multi-omics analyses have identified oncogenic piR-1170, which is specifically enriched in TNBC brain lesions, and its overexpression correlates with unfavorable patient prognosis (219). Moreover, chemotherapy-induced resistance signatures exhibit subtype-specific patterns: in mesenchymal-like TNBC cells, treatment leads to upregulation of macrophage colony-stimulating factor (M-CSF) and CXCL8/IL-8, whereas epithelial TNBC cells show upregulation of CCL2/MCP-1 (220).

These subtypes of TNBC are enriched in specific molecular pathways, gene expressions, and gene mutations, suggesting that chemokine expression may also vary among the different subtypes. Indeed, the BLIA subtype exhibited a significant enrichment of CXCL9, CXCL11, CXCL10, and CXCL13 compared with other types, and is associated with a more favorable clinical outcome (212). Thus, while chemokine targets are promising, the lack of validated subtype-specific biomarkers hinders personalized therapy. Future translational efforts should prioritize tailoring strategies to tumor molecular subtypes and integrating multi-omics data to guide clinical decision-making.

### 5.3 Limited clinical research and availability in patients

While preclinical studies have indicated promising outcomes in targeting chemokines for the treatment of TNBC, clinical evidence remains limited, and the efficacy of these therapies requires further validation through clinical trials. As previously mentioned, the majority of clinical trials are currently in phase I or II, with few results being publicly available (172). As summarized in **Table 2**, some clinical trials have been terminated or withdrawn. This could be due to various reasons like severe side effects, insufficient funding, or poor results, however, the conducting institution has not released an official statement. Moreover, a significant factor is the economic burden associated with these therapies, as the available drugs are almost exclusively monoclonal antibodies that may be too costly for patients to afford.

### 5.4 The future of chemokine targets

In TNBC, chemokines or their receptors play a crucial role in the immune response, as depicted in **Figure 1**. Pharmacological targeting of chemokines could enhance the immune response, leading to improved clinical outcomes (87). Furthermore, the expression level of chemokines could serve as biomarkers for patient diagnosis or prognosis (148), offering more specific molecular information and potentially leading to individualized therapy. Advances in artificial intelligence and multi-omics integration are enabling the development of predictive models for immunotherapy response, thereby enhancing the clinical utility of chemokine-based biomarkers (218). Given the inherent complexity of the chemokine network within tumors, combinatorial strategies may prove to be a more promising strategy for treating TNBC patients. As summarized in **Table 2**, most clinical trials use chemokine targeted reagents in combination with cytotoxic drugs, such as Carboplatin, Eribulin and Paclitaxel, as a therapeutic method (172). This trend suggests that combination therapies might offer superior efficacy compared with monotherapy in the future clinical practice. Beyond conventional drug combinations, emerging strategies include nanoparticle-based co-delivery of chemokine inhibitors with immunomodulators, bispecific antibodies targeting chemokine receptors and immune checkpoints, and oncolytic virus-mediated chemokine delivery to remodel the TME (221, 222). Moreover, the progress in intrinsic subtyping of TNBC through gene expression analysis has provided more possibilities for targeting chemokines. For instance, the mesenchymal subtype identified by Lehmann et al. is associated with elevated levels of TGF-β, mTOR, Rac1/Rho, Wnt/β-catenin, FGFR, PDGFR, and VEGF signaling pathways, which are crucial for the EMT process and the proliferation of CD44^+^ CD24^-^ stem cells (213, 223, 224). As described above, CCL20, CXCR1, CCL2, CXCL8, and other molecules play a crucial role in regulating the function of CSCs (53, 92–94), and targeting these chemokines might be more efficient for treating the TNBC. In contrast, CXCL10 exerts anti-tumor immune effects. Existing studies have validated that upregulated CXCL10 markedly boosts tumor infiltration of DCs and strengthens the presentation of tumor-associated antigens to T lymphocytes, thereby eliciting robust specific anti-tumor immune responses (78). Accordingly, combined specific targeting of multiple factors may bring promising therapeutic benefits for TNBC treatment.

As clinical trials continue to advance, an increasing number of outcomes are being disseminated through public registries and scientific conferences, offering more evidence for chemokine-targeted therapeutic strategies. Furthermore, various preclinical targets may be proposed for clinical trials in the future, allowing for further validation of their efficacy. Collectively, there are growing opportunities for treating TNBC through the targeting of chemokines.

## 6 Conclusion

TNBC, the most aggressive subtype of breast cancer, is characterized by high rates of recurrence and distant metastasis, as well as poor overall survival. The current lack of targeted therapies for this disease underscores a critical unmet clinical need. Chemokines, small proteins that orchestrate leukocyte migration, activation, and differentiation, play an essential role in immune regulation and have been identified as potential targets for therapeutic intervention in cancers, including TNBC. Decades of research efforts have facilitated the development of numerous novel chemokine-targeting agents, such as Leronlimab, CM369, Reparixin, and others. Encouragingly, several of these agents have advanced into clinical trials, with some of these trials have yielded promising results. Nevertheless, several challenges remain to be addressed, including the complexity of chemokine networks, the multifaceted nature of the TME, the marked heterogeneity of TNBC, and the currently limited clinical evidence base. Looking forward, it is becoming increasingly clear that the successful therapeutic strategies of TNBC will concentrate on developing immune response targeting, precise TNBC subtyping, and combination therapeutic strategies.

## Glossary

HER-2: Human epidermal growth factor receptor 2
TNBC: Triple-negative breast cancer
NCCN: National Comprehensive Cancer Network
PD-L1: Programmed cell death ligand 1
PARP: Poly ADP-ribose polymerase
ADC: Antibody-drug conjugate
IL-8: Interleukin-8
RANTES: Regulated on activation, normal T expressed and secreted
MIP-2: Macrophage inflammatory protein-2
FDA: Food and drug administration
HIV: Human immunodeficiency virus
miRNAs: microRNAs
TIME: Tumor immune microenvironment
NC-IUPHAR: Nomenclature and Standards Committee of the International Union of Basic and Clinical Pharmacology
GPCRs: G protein-coupled receptors
ACKRs: Atypical chemokine receptors
CSCs: Cancer stem cells
TAMs: Tumor-associated macrophages
Tregs: Regulatory T cells
MDSCs: Myeloid-derived suppressor cells
CAFs: Cancer-associated fibroblasts
EVs: Extracellular vesicles
EMT: Epithelial-mesenchymal transition
TME: Tumor microenvironment
NK: Natural killer
DCs: Dendritic cells
apCAFs: Antigen-presenting CAFs
Bregs: Regulatory B cells
cDC1s: Type 1 conventional dendritic cells
ICB: Immune checkpoint blockade
TADCs: Tumor-associated DCs
JNK/c-Jun: C-Jun N-terminal Kinase
IFN-γ: Interferon-gamma
IMCs: Immunosuppressive myeloid cells
BCSCs: Breast cancer stem cells
CAAs: Cancer-associated adipocytes
ECs: Endothelial cells
ECFCs: Endothelial colony-forming cells
TANs: Tumor-associated neutrophils
TRAIL: Tumor necrosis factor-related apoptosis-inducing ligand
MMP9: Matrix metallopeptidase 9
FOLR1: Folate receptor alpha
MMAE: Monomethyl auristatin E
scFv: Single-chain variable fragment
DPC: Dextrin-PEI-CM nano-complex
CM: Cyclam monomer
TGF-β: Transforming growth factor-β
siRNA: Small interfering RNA
shRNA: Short hairpin RNA
TAT: Trans-activating transcriptor
PGG: Pentagalloyl glucose
OS: Overall survival
ADCC: Antibody dependent cell-mediated cytotoxicity
ADCP: Antibody dependent cellular phagocytosis
PFS: Progression-free survival
IDO1: Indoleamine 2,3-dioxygenase 1
M-CSF: Macrophage colony-stimulating factor
